# Association of remnant cholesterol with cognitive impairment: a cross-sectional study

**DOI:** 10.3389/fnhum.2026.1771503

**Published:** 2026-02-03

**Authors:** Weili Bai, Yong He, Xuewen Xiao, Tieshi Zhu

**Affiliations:** 1 Department of Neurology, Liuyang Jili Hospital, Changsha, Hunan, China; 2 Department of Neurology, Xiangya Hospital, Central South University, Changsha, China; 3 Department of Neurology, Zhanjiang Central Hospital, Guangdong Medical University, Zhanjiang, Guangdong, China

**Keywords:** cognitive impairment, cross-sectional study, machine learning, remnant cholesterol, rural areas

## Abstract

**Background:**

Although dyslipidemia has been implicated in the development of cognitive impairment, the association between remnant cholesterol (RC) and cognitive outcomes remains incompletely understood.

**Methods:**

We conducted a cross-sectional study using data from 1,136 participants at Liuyang Jili Hospital from 2022. RC was calculated as total cholesterol minus low-density and high-density lipoprotein cholesterol, and cognitive function was assessed using the Mini-Mental State Examination. Restricted cubic spline and multivariable logistic regression models were used to evaluate associations between RC and cognitive impairment, with subgroup, and sensitivity analyses. Machine learning models with SHapley additive explanation (SHAP) values were applied to assess variable importance.

**Results:**

Participants had a median age of 68 years, and 21.2% had cognitive impairment. Higher RC levels were associated with increased risk of cognitive impairment (per 1-mmol/L increase: adjusted odds ratio, 3.47; 95% CI, 2.34–5.25; *P* < 0.01), with no evidence of a non-linear relationship. The association remained consistent across subgroups. In machine learning analyses, RC ranked third in variable importance after waist circumference, and body mass index, and SHAP dependency plots demonstrated a monotonic positive relationship between RC and cognitive impairment risk.

**Conclusion:**

Elevated RC levels are positively associated with an increased risk of cognitive impairment. These findings suggest that remnant cholesterol may be an important and modifiable risk factor for cognitive dysfunction, warranting confirmation in prospective longitudinal studies.

## 1 Introduction

Cognitive impairment represents a significant global public health concern, imposing a substantial burden on individuals and society alike (Deng et al., 2021; GBD Dementia Forecasting Collaborators, 2022; Xu et al., 2024). Worldwide, the number of dementia-related deaths rose from 0.56 million in 1990 to 1.62 million in 2019, and this figure is projected to reach 4.91 million by 2050 (Li Z. et al., 2024). In China, an estimated 16.99 million individuals are affected by Alzheimer disease and other dementias, with a high prevalence among the older population (Huang et al., 2025; Wu et al., 2024; Zhang et al., 2025). Moreover, recent evidence indicates that the incidence, prevalence, and mortality of dementia in China have all increased over time, imposing a substantial economic burden on families, society, and the health care system (Zhi et al., 2025). Despite ongoing advances, effective treatments for dementia remain limited (Reuben et al., 2024). Accordingly, the identification of modifiable risk factors for cognitive impairment is of considerable clinical and public health relevance.

A growing body of evidence suggests that lipid profiles, including low-density lipoprotein cholesterol (LDL), and triglycerides (TG), are important risk factors for cognitive impairment, with elevated levels associated with an increased risk of cognitive decline (Ferguson et al., 2023; Juul Rasmussen et al., 2024; Pan et al., 2024; Shang et al., 2024). Remnant cholesterol (RC), the cholesterol content of triglyceride-rich lipoproteins, has recently gained attention as a risk factor for cardiovascular disease and has been strongly linked to both stroke and coronary heart disease (Chen et al., 2024; Sturzebecher et al., 2023; Wadstrom et al., 2023). Although elevated RC has been associated with stroke risk, current evidence linking RC to cognitive impairment is limited, with heterogeneous findings across studies (Xiong et al., 2024). For instance, a study by Heo et al. reported a positive association between higher RC levels and increased risk of dementia (Heo et al., 2024). Conversely, another study observed a more complex pattern, with the risk of cognitive impairment initially decreasing, then increasing with rising RC levels (Liu et al., 2024). These inconsistencies underscore the need for further investigation. The present study aims to explore the association between RC and cognitive impairment using data from Liuyang Jili Hospital, and to evaluate the relative contribution of RC to cognitive impairment prediction by applying machine learning algorithms combined with SHAP analysis.

## 2 Methods

### 2.1 Population

The study population consisted of 1,136 individuals who underwent medical examinations and completed the Mini-Mental State Examination (MMSE) at the Medical Examination Center of Liuyang Jili Hospital in 2022. The median age was 68 years, and 54.0% of participants were women.

### 2.2 Inclusion and exclusion criteria

Participants were included in the study if they met the following criteria: (1) age >40 years; (2) underwent a routine health examination at the Medical Examination Center of Liuyang Jili Hospital; (3) completed the MMSE during the same visit; and (4) had complete lipid profile data, including total cholesterol (TC), high-density lipoprotein cholesterol (HDL), and LDL. Exclusion criteria were: (1) failure to complete the MMSE assessment; (2) missing or incomplete lipid profile data; (3) Severe systemic illnesses (e.g., end-stage renal disease, liver failure) that may confound the association between lipids and cognition.

### 2.3 Outcome

In this study, cognitive function was assessed using the MMSE (Jia et al., 2021). Classification thresholds were based on participants' educational attainment. Cognitive impairment, including both MCI and dementia, was defined as an MMSE score of ≤ 17 for individuals with no formal education, ≤ 19 for those with a primary school education, and ≤ 24 for those with a junior high school education or higher (He et al., 2025). All cognitive assessments were administered by trained neurologists, psychiatrists, or nurses to ensure accuracy and consistency.

### 2.4 Exposure

RC, expressed in mmol/L, was calculated using the following formula (Li M. et al., 2024; Yao and Yang, 2024): RC = TC - HDL – LDL. All blood samples were collected in the morning between 8:00 and 10:00 a.m. after overnight fasting, as part of routine health examinations.

### 2.5 Covariates

Covariates included age, sex, body mass index (BMI), TC, LDL, HDL, educational level, smoking status, alcohol consumption, diabetes, hypertension, and ischemic stroke. BMI was calculated based on height and weight measured at the Health Checkup Center. Information on educational level, smoking, alcohol consumption, diabetes, hypertension, and ischemic stroke was obtained through self-report. Laboratory measurements of TC, LDL, and HDL were provided by the Department of Laboratory Medicine at Liuyang Jili Hospital.

### 2.6 Statistics

All statistical analyses were conducted using *R* software, version 4.4.1 (*R* Foundation for Statistical Computing). Baseline characteristics were compared using the Kruskal–Wallis test for continuous variables and the χ^2^ test for categorical variables. Continuous variables are presented as median (interquartile range [IQR]), and categorical variables as number (percentage).

Restricted cubic spline (RCS) analyses with three knots placed at the 10th, 50th, and 90th percentiles of the RC distribution were used to evaluate potential non-linear associations between RC and cognitive impairment, as this knot placement is commonly adopted to balance model flexibility and stability while minimizing the risk of overfitting. Logistic regression models were applied to assess the association between RC and cognitive impairment. Four multivariable models were constructed: Model 1 was unadjusted; Model 2 was adjusted for age, sex, waist circumference, and body mass index (BMI); Model 3 was further adjusted for educational level, smoking status, alcohol consumption, HDL, and LDL; and Model 4 additionally included hypertension, diabetes, and ischemic stroke. Sensitivity analyses were conducted by excluding participants with a history of ischemic stroke and by calculating *E*-values to assess the robustness of the observed associations to potential unmeasured confounding. Covariates included in the RCS analyses were consistent with those in Model 4. Multicollinearity was evaluated using the variance inflation factor (VIF), with VIF > 5 indicating collinearity. All variables included in the models had VIF < 5, suggesting no evidence of multicollinearity (Supplementary Table 1). Two-sided *P* values < 0.05 were considered statistically significant. Subgroup analyses were conducted, with each model adjusted for the same covariates as model 4, excluding the stratifying variable.

The study population (*n* = 1,136) was randomly divided into a training set (70%) and a test set (30%). In the training set, the variables included in model 4 were used to develop multiple machine learning models, with 5-fold cross-validation. The algorithms evaluated included Random Forest, Gradient Boosting, Support Vector Machine (kernel-based), Logistic Regression, k-Nearest Neighbor, Partial Least Squares, Adaptive Boosting, Neural Network, Linear Discriminant Analysis, Lasso Regression, CATBoost, and LightGBM. For the CatBoost model, training was performed using default hyperparameter settings, and 5-fold cross-validation was applied to assess model performance, and reduce the risk of overfitting. The model with the best performance was selected, and SHapley Additive exPlanations (SHAP) analysis was applied to quantify the contribution of each variable to cognitive impairment prediction. Potential synergistic effects of other variables on the association between RC and cognitive impairment were also examined.

## 3 Results

### 3.1 1 Baseline characteristics of the study population

Table 1 summarizes the baseline characteristics of 1,136 participants, including 895 without cognitive impairment and 241 with cognitive impairment. No significant differences were observed between groups in age, sex, BMI, LDL, hypertension, or diabetes (all *P* > 0.05). Participants with cognitive impairment had lower MMSE scores, a higher proportion with elementary school or higher education, greater waist circumference, a higher prevalence of non-smoking, and non-drinking status, higher TC, and RC levels, lower HDL levels, and a higher prevalence of ischemic stroke history (all *P* < 0.05) (Table 1).

**Table 1:** Baseline characteristics of participants stratified by cognitive impairment status.

**Variables**	**Total *N* = 1,136**	**No *N* = 895**	**Yes *N* = 241**	***P***
Age (year)	68.0 (66.0, 72.0)	68.0 (66.0, 72.0)	69.0 (66.0, 74.0)	0.086
Female	613 (54.0%)	474 (53.0%)	139 (57.7%)	0.218
MMSE	25.0 (21.0, 28.0)	26.0 (24.0, 28.0)	19.0 (15.0, 23.0)	< 0.001
Education				< 0.001
>Elementary school	341 (30.0%)	236 (26.4%)	105 (43.6%)	
Elementary school	599 (52.7%)	501 (56.0%)	98 (40.7%)	
Illiterate	196 (17.3%)	158 (17.7%)	38 (15.8%)	
Waist (cm)	80.0 (74.3, 86.0)	79.0 (74.0, 84.7)	83.0 (77.0, 89.0)	< 0.001
BMI (kg/m^2^)	24.4 (22.3, 26.3)	24.4 (22.3, 26.2)	24.7 (22.1, 26.6)	0.599
Smoke				0.037
Current	213 (18.8%)	181 (20.2%)	32 (13.3%)	
Former	52 (4.58%)	38 (4.25%)	14 (5.81%)	
Never	871 (76.7%)	676 (75.5%)	195 (80.9%)	
Drink				0.025
Every day	40 (3.52%)	30 (3.35%)	10 (4.15%)	
Never	1,026 (90.3%)	801 (89.5%)	225 (93.4%)	
Sometime	70 (6.16%)	64 (7.15%)	6 (2.49%)	
TC (mmol/L)	5.07 (4.32, 5.77)	5.01 (4.30, 5.74)	5.20 (4.44, 5.85)	0.045
LDL (mmol/L)	2.96 (2.36, 3.54)	2.95 (2.37, 3.52)	3.05 (2.34, 3.59)	0.794
HDL (mmol/L)	1.44 (1.23, 1.66)	1.45 (1.24, 1.68)	1.37 (1.20, 1.60)	0.006
RC (mmol/L)	0.61 (0.32, 0.87)	0.56 (0.29, 0.83)	0.79 (0.55, 1.02)	< 0.001
Hypertension	562 (49.5%)	432 (48.3%)	130 (53.9%)	0.136
Diabetes	171 (15.1%)	131 (14.6%)	40 (16.6%)	0.513
Ischemic stroke	21 (1.85%)	10 (1.12%)	11 (4.56%)	0.001

Data are presented as median (Q1, Q3) or *n* (%). Q1, 1st Quartile; Q3, 3st Quartile; MMSE, Mini-Mental State Examination; BMI, body mass index; TC, total cholesterol; LDL, low-density lipoprotein cholesterol; HDL, high-density lipoprotein cholesterol; RC, remnant cholesterol.

### 3.2 Elevated RC is positively associated with the risk of cognitive impairment

**Figure 1:**
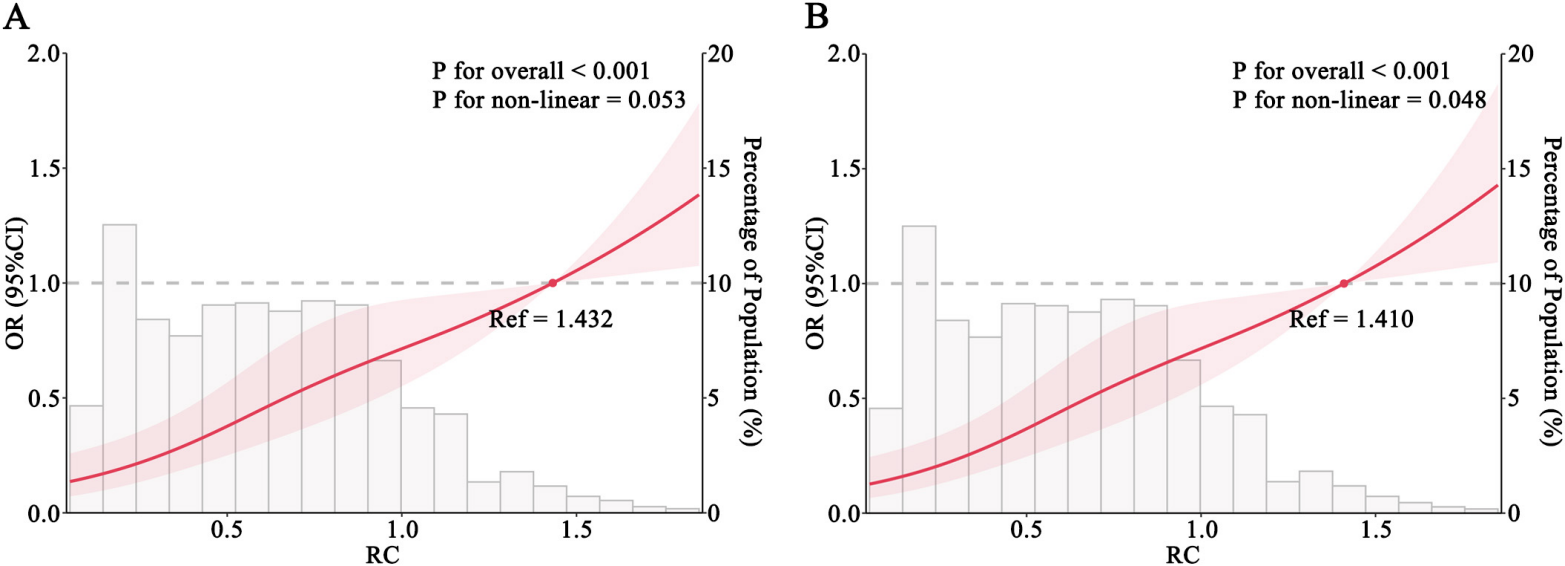
Restricted cubic spline analysis of the association between rc and cognitive impairment. **(A)** Full study population. **(B)** Population excluding individuals with a history of ischemic stroke. Solid red lines indicate OR and shaded areas indicate 95% CI. Histograms represent the distribution of RC in the corresponding population.

**Figure 2:**
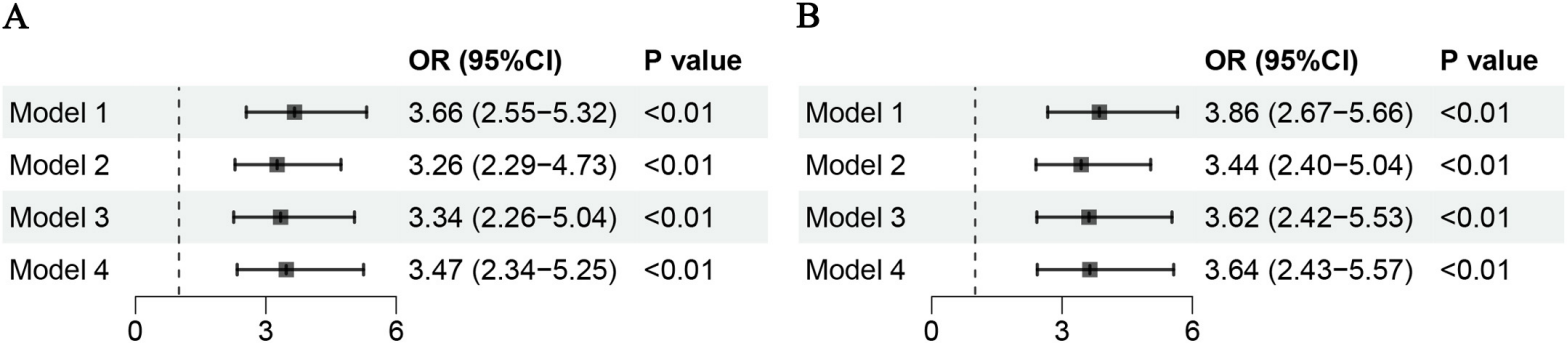
Logistics analysis of the association between rc and cognitive impairment. **(A)** Full study population. **(B)** Population excluding individuals with a history of ischemic stroke. ORs and 95% CIs are shown for each subgroup. Model 1 was unadjusted. Model 2 was adjusted for age, sex, waist circumference, and BMI. Model 3 was further adjusted for educational level, smoking status, alcohol consumption, HDL, and LDL. Model 4 additionally included hypertension and diabetes; in **(B)**, history of ischemic stroke was not included as a covariate because participants with this condition were excluded.

**Figure 3:**
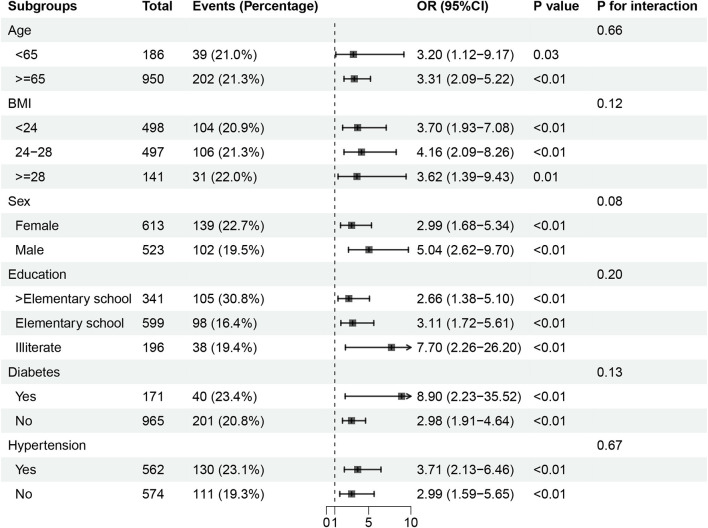
Subgroup analyses of the association between rc and cognitive impairment. ORs and 95% CIs are shown for each subgroup. Models were adjusted for the same covariates as in model 4, excluding the stratifying variable for each subgroup.

RCS analysis demonstrated a positive association between higher RC levels and increased risk of cognitive impairment, without evidence of a non-linear relationship (*P* for overall < 0.001; *P* for non-linearity = 0.053) (Figure 1A). In logistic regression models, elevated RC was consistently associated with higher odds of cognitive impairment across models 1 through 4 (Figure 2A). The *E*-value for the observed association between RC and cognitive impairment was 3.24, with a lower confidence limit *E*-value of 2.49, suggesting that substantial unmeasured confounding would be required to explain away the observed association. Sensitivity analyses excluding participants with a history of ischemic stroke yielded similar results (Figures 1B, 2B). In subgroup analyses, higher RC levels were positively associated with cognitive impairment risk across all subgroups; however, some subgroups with smaller sample sizes had wider confidence intervals, although the associations remained statistically significant (*P* < 0.05) (Figure 3).

### 3.3 Variable importance and interaction effects of RC in machine learning models

**Figure 4:**
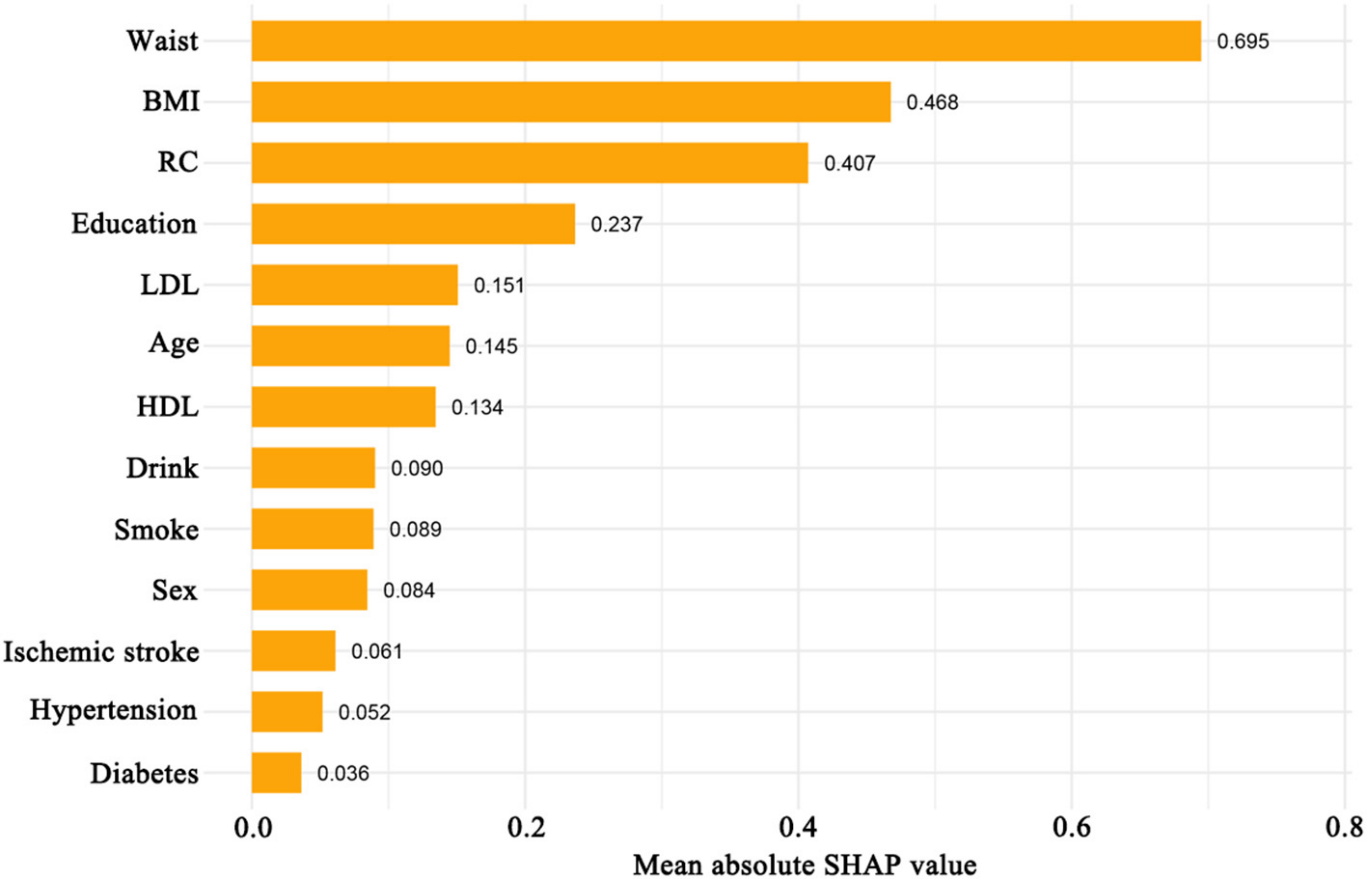
Variable Importance Ranking for Cognitive Impairment Based on SHAP Values From the CatBoost Model. Mean absolute SHAP values are shown for variables included in the CatBoost machine learning model. Higher SHAP values indicate greater contribution to the model's prediction of cognitive impairment.

**Figure 5:**
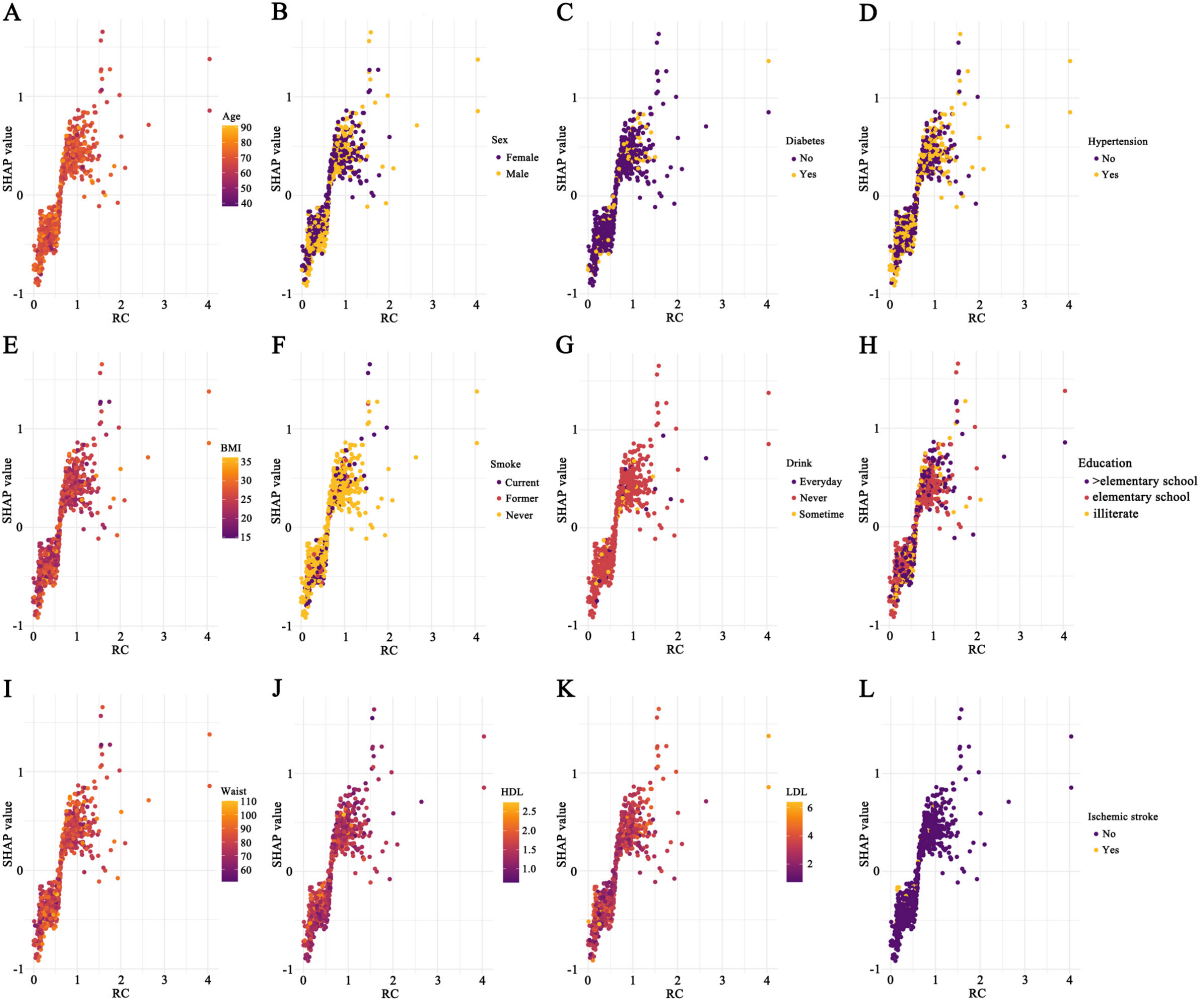
SHAP dependency plots for the association between rc and cognitive impairment. **(A–L)** Show SHAP dependency plots of RC for cognitive impairment across subgroups defined by age **(A)**, sex **(B)**, diabetes **(C)**, hypertension **(D)**, BMI **(E)**, smoke **(F)**, drink **(G)**, educational **(H)**, waist circumference **(I)**, HDL **(J)**, LDL **(K)**, and history of ischemic stroke **(L)**. Each point represents an individual participant, with color gradients indicating the corresponding subgroup variable value. Positive SHAP values indicate increased predicted risk of cognitive impairment.

The study population (*n* = 1,136) was randomly divided into a training set (70%) and a test set (30%) for machine learning model development (Supplementary Table 2). The CatBoost model demonstrated the best performance among the tested algorithms (Supplementary Figures 1A, B; Supplementary Figures 2A, B). SHAP analysis based on the CatBoost model identified waist circumference and BMI as the variables with the highest SHAP values, followed by RC (SHAP value, 0.407) (Figure 4). SHAP dependency plots indicated a monotonic positive association between RC and the risk of cognitive impairment, with the most pronounced risk increase observed at RC levels of approximately 0.8 to 1.5 mmol/L. Interaction analyses showed that advanced age, diabetes, hypertension, greater waist circumference, lower HDL, higher LDL, and history of ischemic stroke amplified the association between RC, and cognitive impairment risk, whereas the modifying effects of smoking status, alcohol consumption, and educational attainment were weaker (Figures 5A–L).

## 4 Discussion

In this study, analysis of data from Liuyang Jili Hospital demonstrated that higher RC levels were positively associated with an increased risk of cognitive impairment. This association remained significant after adjustment for multiple confounding variables and across diverse subgroups. Machine learning analyses using SHAP values indicated that RC was the third most influential variable for cognitive impairment risk, following waist circumference and body mass index. SHAP dependency analysis further confirmed a positive, monotonic association between RC and cognitive impairment.

Several studies have examined the association between RC and cognitive function, with most reporting positive associations. The largest investigation, conducted by Heo et al., analyzed data from 26 215 960 Korean individuals (median follow-up, 10.3 years) and found that higher RC levels were significantly associated with increased risks of all-cause dementia, Alzheimer disease (AD) dementia, and vascular dementia. Comparing the highest with the lowest RC quartile, fully adjusted hazard ratios (HRs) were 1.11 (95% CI, 1.09–1.13) for all-cause dementia, 1.11 (95% CI, 1.09–1.13) for AD dementia, and 1.15 (95% CI, 1.09–1.21) for vascular dementia (Heo et al., 2024). Most other studies, including the present analysis, have been cross-sectional. Ai et al. analyzed 1,007 individuals in Wuhan, China, and reported that RC was significantly associated with amnestic mild cognitive impairment (Ai et al., 2024). Two additional studies based on data from the U. S. National Health and Nutrition Examination Survey (NHANES) further explored this association. Xie et al. found that each 1 mmol/L increase in RC was associated with lower odds of achieving a high total Consortium to Establish a Registry for Alzheimer's Disease (CERAD) score, with an adjusted OR of 0.74 (95% CI, 0.58–0.94) (Xie et al., 2022). In contrast, Liu et al., using the same NHANES dataset, observed a more complex pattern. The risk of cognitive impairment initially decreased, then increased, and finally increased again with rising RC levels; however, the proportion of cognitive impairment was ultimately higher among individuals with elevated RC (Liu et al., 2024). Findings from the present study align with most prior investigations, demonstrating a positive association between RC and cognitive impairment without the complex non-linear relationship reported by Liu et al. Unlike previous NHANES-based studies conducted in the U.S. population, the present study was based on a rural Chinese cohort, characterized by different socioeconomic conditions and health profiles, which extends the existing evidence to a non-Western population. SHAP dependency plots in our analysis showed consistently higher SHAP values for elevated RC levels compared with lower levels. In addition, our machine learning–based variable importance ranking identified RC as the third most influential factor for cognitive impairment risk, after waist circumference and body mass index—a finding not previously reported. Because the large cohort analysis by Heo et al. was retrospective and the present and other studies were cross-sectional, reverse causality cannot be excluded. Prospective longitudinal studies are needed to clarify the temporal and causal relationship between RC and cognitive outcomes.

Subgroup analyses showed higher ORs for cognitive impairment in participants aged ≥65 years, men, and those with diabetes or hypertension. Although the *P* values for interaction were < 0.05, the findings highlight the need to consider the potential impact of RC on cognition among individuals with multiple cardiovascular risk factors. In SHAP dependency plots, comparable RC levels were associated with higher SHAP values in the presence of these risk factors. A similar phenomenon was reported in the large cohort study by Heo et al., suggesting possible synergistic effects between RC and cardiovascular risk factors; however, further research is needed to confirm this interaction.

The biological mechanisms underlying the association between RC and cognitive function are not yet fully understood, but may be related to atherosclerosis. Numerous studies have shown that elevated RC promotes the development of atherosclerosis, which can reduce cerebral blood perfusion and increase the risk of stroke (Han et al., 2024; Li et al., 2022; Pinto et al., 2023). These factors are known contributors to vascular dementia, as supported by findings from Heo et al. (Heo et al., 2024; Mok et al., 2024). Additionally, several studies utilized cognitive test scales that assess executive function, which is often impaired in the early stages of vascular dementia. This indirectly suggests a potential link between elevated RC and vascular cognitive impairment (Liu et al., 2024; Yao and Yang, 2024). However, Heo et al. also found that higher RC levels were associated with an increased risk of AD, indicating that other, non-vascular mechanisms may also be involved—warranting further investigation.

This study has several limitations. First, its retrospective cross-sectional design precludes causal inference; only associations can be reported. Second, although adjustments were made for multiple confounding variables, the possibility of residual confounding cannot be excluded. Third, lipid levels were measured only once, which may not reflect long-term exposure, as lipid profiles can fluctuate over time; repeated measurements would provide more reliable estimates. Finally, cognitive function was assessed using the MMSE, a multidimensional screening tool; however, a single scale may capture only certain aspects of cognitive function and may not fully reflect the complexity of cognitive impairment, particularly in domains such as executive function.

## 5 Conclusion

RC was positively associated with the risk of cognitive impairment, and this association persisted across multiple subgroups. These findings underscore the potential importance of RC as a risk factor for cognitive impairment. Given that RC is routinely available and easily calculated from standard lipid profiles, it may have practical value for clinical risk assessment. However, prospective longitudinal studies are warranted to confirm these associations and clarify causality.

## Data Availability

The datasets presented in this article are not readily available because the data used in this study were obtained from the Liuyang Jili Hospital Medical Examination Center, and their use was contingent upon obtaining ethical approval from the hospital. Without this approval, the data could not be used, and therefore cannot be made publicly available. The authors do not have permission to share data. Requests to access the datasets should be directed to Yong He, 277475748@qq.com.

## Supplementary material

Supplementary Figure S1 Receiver operating characteristic curves of machine learning models for predicting cognitive impairment. **(A)** Training set. **(B)** Test set. Models include random forest, gradient boosting, support vector machine (SVM, kernel-based), logistic regression, k-nearest neighbor, partial least squares (PLS), adaptive boosting, neural network, linear discriminant analysis, lasso regression, CatBoost, and LightGBM. Area under the curve (AUC) values are indicated in the legend.

Supplementary Figure S2 Decision curve analysis of machine learning models for predicting cognitive impairment. **(A)** Training set. **(B)** Test set. The y-axis represents net benefit, and the x-axis represents threshold probability. Models include random forest, gradient boosting, support vector machine (SVM, kernel-based), logistic regression, k-nearest neighbor, partial least squares (PLS), adaptive boosting, neural network, linear discriminant analysis, lasso regression, CatBoost, and LightGBM. “Treat all” and “treat none” strategies are shown as reference lines.
