# Supplementary material for: Association of remnant cholesterol with cognitive impairment: a cross-sectional study
Source: Front Hum Neurosci. 2026 Feb 3;20:1771503. doi: 10.3389/fnhum.2026.1771503 (PMC12909580; doi:10.3389/fnhum.2026.1771503)
Supplement: Supplementary file 1 [file Table_1.docx]

Table S1. Assessment of Multicollinearity Using VIF and Tolerance

|  | | VIF | Tolerance |
| --- | --- | --- | --- |
| RC | | 1.154 | 0.867 |
| Age | | 1.077 | 0.929 |
| Sex | Female | ref | ref |
|  | Male | 1.619 | 0.618 |
| Education | >elementary school | ref | ref |
|  | elementary school | 1.209 | 0.827 |
|  | illiterate | 1.243 | 0.805 |
| Waist | | 3.677 | 0.272 |
| BMI | | 3.515 | 0.284 |
| Smoke | current | ref | ref |
|  | former | 1.399 | 0.715 |
|  | never | 1.844 | 0.542 |
| Drink | everyday | ref | ref |
|  | never | 1.936 | 0.517 |
|  | sometime | 1.858 | 0.538 |
| Hypertension | no | ref | ref |
|  | yes | 1.053 | 0.950 |
| Diabetes | no | ref | ref |
|  | yes | 1.066 | 0.938 |
| Ischemic stroke | no | ref | ref |
|  | yes | 1.038 | 0.964 |
| LDL | | 1.186 | 0.843 |
| HDL | | 1.285 | 0.778 |

VIF, variance inflation factor; RC, remnant cholesterol; BMI, body mass index; LDL, low-density lipoprotein cholesterol; HDL, high-density lipoprotein cholesterol.
