# Supplementary material for: Association of remnant cholesterol with cognitive impairment: a cross-sectional study
Source: Front Hum Neurosci. 2026 Feb 3;20:1771503. doi: 10.3389/fnhum.2026.1771503 (PMC12909580; doi:10.3389/fnhum.2026.1771503)
Supplement: Supplementary file 2 [file Table_2.docx]

Table S2. Baseline Characteristics of Participants in the Training and Test Sets

|  | Total  N=1136 | Test  N=360 | Train  N=796 | ***P*** |
| --- | --- | --- | --- | --- |
| Age (year) | 68.0 (66.0,72.0) | 69.0 (66.0,72.0) | 68.0 (66.0,72.0) | 0.662 |
| Female | 613 (54.0%) | 179 (52.6%) | 434 (54.5%) | 0.606 |
| Cognitive impairment | 241 (21.2%) | 72 (21.2%) | 169 (21.2%) | 1.000 |
| MMSE | 25.0 (21.0,28.0) | 25.0 (22.0,28.0) | 25.0 (21.0,28.0) | 0.879 |
| Education |  |  |  | 0.913 |
| >elementary school | 341 (30.0%) | 104 (30.6%) | 237 (29.8%) |  |
| elementary school | 599 (52.7%) | 176 (51.8%) | 423 (53.1%) |  |
| illiterate | 196 (17.3%) | 60 (17.6%) | 136 (17.1%) |  |
| Waist (cm) | 80.0 (74.3,86.0) | 80.0 (74.7,86.0) | 79.3 (74.0,86.0) | 0.730 |
| BMI (kg/m^2^) | 24.4 (22.3,26.3) | 24.4 (22.3,26.2) | 24.4 (22.3,26.4) | 0.741 |
| Smoke |  |  |  | 0.849 |
| current | 213 (18.8%) | 67 (19.7%) | 146 (18.3%) |  |
| former | 52 (4.58%) | 16 (4.71%) | 36 (4.52%) |  |
| never | 871 (76.7%) | 257 (75.6%) | 614 (77.1%) |  |
| Drink |  |  |  | 0.550 |
| everyday | 40 (3.52%) | 12 (3.53%) | 28 (3.52%) |  |
| never | 1026 (90.3%) | 303 (89.1%) | 723 (90.8%) |  |
| sometime | 70 (6.16%) | 25 (7.35%) | 45 (5.65%) |  |
| TC (mmol/L) | 5.07 (4.32,5.77) | 5.00 (4.15,5.74) | 5.09 (4.37,5.77) | 0.301 |
| LDL (mmol/L) | 2.96 (2.36,3.54) | 2.90 (2.30,3.55) | 2.99 (2.41,3.53) | 0.373 |
| HDL (mmol/L) | 1.44 (1.23,1.66) | 1.43 (1.24,1.63) | 1.44 (1.23,1.67) | 0.725 |
| RC (mmol/L) | 0.61 (0.32,0.87) | 0.61 (0.32,0.85) | 0.60 (0.32,0.88) | 0.539 |
| Hypertension | 562 (49.5%) | 165 (48.5%) | 397 (49.9%) | 0.726 |
| Diabetes | 171 (15.1%) | 50 (14.7%) | 121 (15.2%) | 0.902 |
| Ischemic stroke | 21 (1.85%) | 5 (1.47%) | 16 (2.01%) | 0.706 |

Data are presented as median (Q1, Q3) or n (%). Q1, 1st Quartile; Q3, 3st Quartile; MMSE, Mini-Mental State Examination; BMI, body mass index; TC, total cholesterol; LDL, low-density lipoprotein cholesterol; HDL, high-density lipoprotein cholesterol; RC, remnant cholesterol.
