# Supplementary material for: Association of remnant cholesterol with cognitive impairment: a cross-sectional study
Source: Front Hum Neurosci. 2026 Feb 3;20:1771503. doi: 10.3389/fnhum.2026.1771503 (PMC12909580; doi:10.3389/fnhum.2026.1771503)
Supplement: Supplementary file 3 [file Table_3.docx]

Table S3. Performance Metrics of Machine Learning Models for Predicting Cognitive Impairment in the Training Set

| Models | Sensitivity | Specificity | Accuracy | PPV | NPV | F1 | Youden's index |
| --- | --- | --- | --- | --- | --- | --- | --- |
| RandomForest | 0.515 | 0.764 | 0.711 | 0.370 | 0.854 | 0.431 | 0.279 |
| GradientBoosting | 0.438 | 0.834 | 0.750 | 0.416 | 0.846 | 0.427 | 0.272 |
| SVM_Kernel | 0.249 | 0.909 | 0.769 | 0.424 | 0.818 | 0.313 | 0.158 |
| LogisticModel | 0.527 | 0.820 | 0.758 | 0.441 | 0.865 | 0.480 | 0.346 |
| NeighborMethod | 0.420 | 0.718 | 0.655 | 0.286 | 0.821 | 0.341 | 0.138 |
| PLSModel | 0.970 | 0.088 | 0.275 | 0.223 | 0.917 | 0.362 | 0.058 |
| BoostingMethod | 0.426 | 0.841 | 0.753 | 0.419 | 0.845 | 0.422 | 0.267 |
| NeuralNet | 0.527 | 0.809 | 0.749 | 0.426 | 0.864 | 0.471 | 0.335 |
| DiscriminantModel | 0.450 | 0.834 | 0.753 | 0.422 | 0.849 | 0.436 | 0.284 |
| Lasso | 0.420 | 0.864 | 0.770 | 0.455 | 0.847 | 0.437 | 0.285 |
| CATBoost | 0.817 | 0.971 | 0.938 | 0.885 | 0.952 | 0.849 | 0.788 |
| LightGBM | 0.988 | 0.971 | 0.975 | 0.903 | 0.997 | 0.944 | 0.959 |

PPV, positive predictive value; NPV, negative predictive value; F1, F1 score.
