# Supplementary material for: Association of remnant cholesterol with cognitive impairment: a cross-sectional study
Source: Front Hum Neurosci. 2026 Feb 3;20:1771503. doi: 10.3389/fnhum.2026.1771503 (PMC12909580; doi:10.3389/fnhum.2026.1771503)
Supplement: Supplementary file 4 [file Table_4.docx]

Table S4. Performance Metrics of Machine Learning Models for Predicting Cognitive Impairment in the Test Set

| Models | Sensitivity | Specificity | Accuracy | PPV | NPV | F1 | Youden's index |
| --- | --- | --- | --- | --- | --- | --- | --- |
| RandomForest | 0.514 | 0.784 | 0.726 | 0.389 | 0.857 | 0.443 | 0.297 |
| GradientBoosting | 0.486 | 0.843 | 0.768 | 0.455 | 0.859 | 0.470 | 0.329 |
| SVM_Kernel | 0.306 | 0.907 | 0.779 | 0.468 | 0.829 | 0.370 | 0.212 |
| LogisticModel | 0.500 | 0.821 | 0.753 | 0.429 | 0.859 | 0.462 | 0.321 |
| NeighborMethod | 0.389 | 0.769 | 0.688 | 0.311 | 0.824 | 0.346 | 0.158 |
| PLSModel | 0.986 | 0.090 | 0.279 | 0.225 | 0.960 | 0.367 | 0.076 |
| BoostingMethod | 0.458 | 0.836 | 0.756 | 0.429 | 0.852 | 0.443 | 0.294 |
| NeuralNet | 0.472 | 0.813 | 0.741 | 0.405 | 0.852 | 0.436 | 0.286 |
| DiscriminantModel | 0.486 | 0.836 | 0.762 | 0.443 | 0.858 | 0.464 | 0.322 |
| Lasso | 0.361 | 0.869 | 0.762 | 0.426 | 0.835 | 0.391 | 0.231 |
| CATBoost | 0.458 | 0.858 | 0.774 | 0.465 | 0.855 | 0.462 | 0.317 |
| LightGBM | 0.458 | 0.881 | 0.791 | 0.508 | 0.858 | 0.482 | 0.339 |

PPV, positive predictive value; NPV, negative predictive value; F1, F1 score.
