# Supplementary figures and images for: Association of remnant cholesterol with cognitive impairment: a cross-sectional study
Source: Front Hum Neurosci. 2026 Feb 3;20:1771503. doi: 10.3389/fnhum.2026.1771503 (PMC12909580; doi:10.3389/fnhum.2026.1771503)

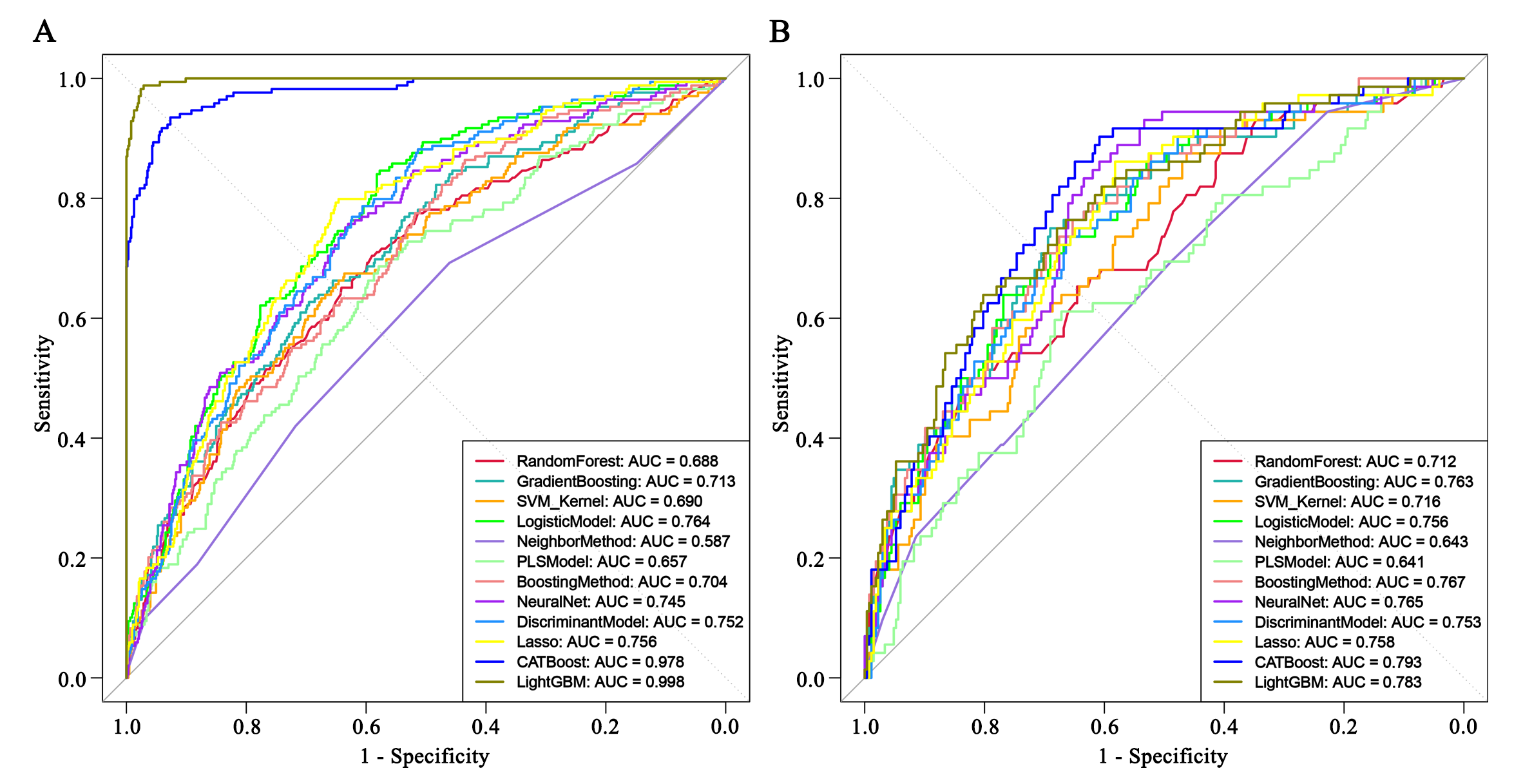

Supplement: Supplementary Figure S1 — Receiver operating characteristic curves of machine learning models for predicting cognitive impairment. (A) Training set. (B) Test set. Models include random forest, gradient boosting, support vector machine (SVM, kernel-based), logistic regression, k-nearest neighbor, partial least squares (PLS), adaptive boosting, neural network, linear discriminant analysis, lasso regression, CatBoost, and LightGBM. Area under the curve (AUC) values are indicated in the legend. [file Image_1.tif]

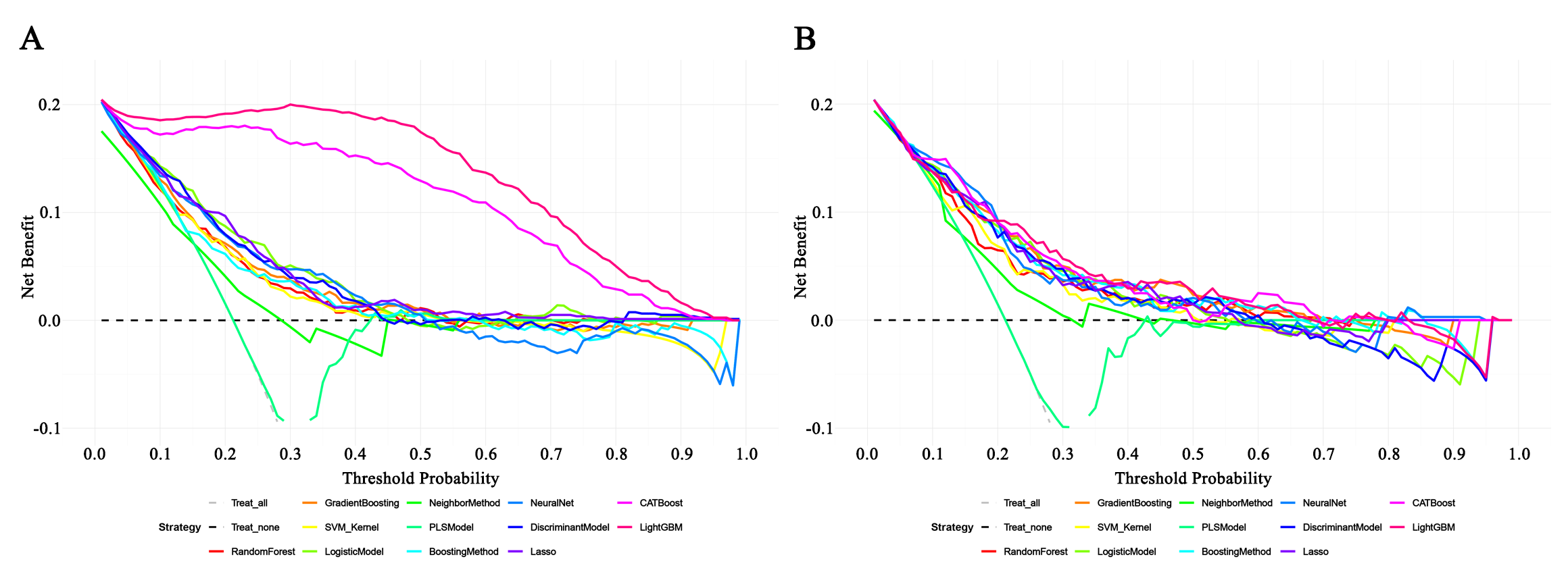

Supplement: Supplementary Figure S2 — Decision curve analysis of machine learning models for predicting cognitive impairment. (A) Training set. (B) Test set. The y-axis represents net benefit, and the x-axis represents threshold probability. Models include random forest, gradient boosting, support vector machine (SVM, kernel-based), logistic regression, k-nearest neighbor, partial least squares (PLS), adaptive boosting, neural network, linear discriminant analysis, lasso regression, CatBoost, and LightGBM. “Treat all” and “treat none” strategies are shown as reference lines. [file Image_2.tif]
